# Guide to Disection

**Published:** 1893-07

**Authors:** 


GUIDE TO DISSECTION OF THE HUMAN BODY,—Comprising improved methods and formulæ of work to be accomplished. By Irving S. Haynes, Ph. B., M. D., Demonstrator of Anatomy in the Medical Department of the University of the city of New York. Member of the Society of the Alumni of Bellevue Hospital, etc., etc. 250 pages, oblong, 12 mo. Pocket edition. Price $1.00. E. B. Treat, Publisher, 5 Cooper Union, New York.

This little work is, as the author states, intended "to supply the beginner with definite directions founded on practical experience in the dissecting room, economize his time by being short and to the point, to fill the gap between the actual dissection of the cadaver and the descriptive anatomy of the standard text books, by giving as clearly as possible the *methods* by which structures are to be exposed; and, above all, it aims to answer this constant question, "What shall I do next?" The purpose of the author as set forth above are well worth the careful thought and attention of any medical man, and the time that

Dr. Haynes has given to this work is labor well bestowed. In our opinion he has attained the objects sought, and has thus "filled a long felt want."

The author divides the work of dissecting the entire body into three courses of three weeks each, taking for the first course the head and neck; second course, the upper extremity and thorax; third course, perineum, male, perineum, female, lower extremity and abdomen. He first gives the line of incision that should be made, and the various structures brought to view by this incision, then how to dissect the different structures so as to see all in their normal relations to each other etc. Altogether it is a work of considerable merit and will be of much assistance to the student in the dissecting room. H.

